# Exposure Is Not Enough: Suppressing Stimuli from Awareness Can Abolish the Mere Exposure Effect

**DOI:** 10.1371/journal.pone.0077726

**Published:** 2013-10-17

**Authors:** Daniel de Zilva, Luke Vu, Ben R. Newell, Joel Pearson

**Affiliations:** School of Psychology, The University of New South Wales, Sydney, New South Wales, Australia; University of California Davis, United States of America

## Abstract

Passive exposure to neutral stimuli increases subsequent liking of those stimuli – the *mere exposure effect.* Because of the broad implications for understanding and controlling human preferences, the role of conscious awareness in mere exposure has received much attention. Previous studies have claimed that the mere exposure effect can occur without conscious awareness of the stimuli. In two experiments, we applied a technique new to the mere exposure literature, called continuous flash suppression, to expose stimuli for a controlled duration with and without awareness. To ensure the reliability of the awareness manipulation, awareness was monitored on a trial-by-trial basis. Our results show that under these conditions the mere exposure effect does not occur without conscious awareness. In contrast, only when participants were aware of the stimuli did exposure increase liking and recognition. Together these data are consistent with the idea that the mere exposure effect requires conscious awareness and has important implications for theories of memory and affect.

## Introduction

Repeated exposure to an initially neutral stimulus increases liking of that stimulus, a phenomenon known as the *mere exposure effect* [1]. The phenomenon has been applied to a wide range of fields, including exposure therapy [2], stereotypes and prejudice [3], brand preferences [4], food preferences [5], and aesthetics [6], [7], [8]. Significant theoretical interest in the phenomenon stems from the important implications that the mere exposure effect has when it occurs without conscious awareness. For example, exposure effects without awareness have been interpreted as evidence for two distinct memory systems, implicit and explicit [9], [10], and as evidence for independence of cognition and affect [11], [12], [13]. However, these interpretations are tenuous because research on the role of awareness in exposure effects has traditionally relied on a single technique – rapid presentation of masked stimuli – which has produced mixed results; sometimes awareness enhances the mere exposure effect [14] and sometimes awareness inhibits it [9].

Evidence of the exposure effect occurring without awareness has been taken from experiments in which masked stimuli are repeatedly presented for a very brief duration (8 ms or less) and are later tested for liking and recognition compared to novel stimuli. Several experiments with this general design have shown that exposure increases the subsequent pleasantness of the stimuli without reliably increasing recognition [10], [11], [12], [15], [16]. Other evidence suggests that awareness might in fact inhibit the mere exposure effect. Bornstein and D′Agostino [9] exposed subjects to some stimuli for 5 ms and others for 500 ms between zero and twenty times. Stimuli exposed for 500 ms were better recognised the more frequently they were exposed, whereas consistent with the above studies, stimuli exposed for 5 ms were no better recognised at high exposure frequencies than low exposure frequencies. Despite this lack of recognition for the 5 ms stimuli, both groups of stimuli were better liked the more frequently they were exposed, and the effect was stronger for stimuli exposed for 5 ms than those exposed for 500 ms. Consistent with previous studies, the authors suggested that awareness is unnecessary for the exposure effect, and that awareness actually inhibits the mere exposure effect.

However, the only similar study comparing stimuli exposed briefly (40 ms) and for long durations (400 ms) has shown a different pattern of liking to Bornstein and D′Agostino [9]. In three experiments, Newell and Shanks [14] showed increased liking for exposed stimuli compared to novel stimuli only at the highest level of exposure frequency and duration, conditions in which recognition performance was greatest. Thus, the masked brief exposure paradigm has produced mixed findings.

**Figure 1:**
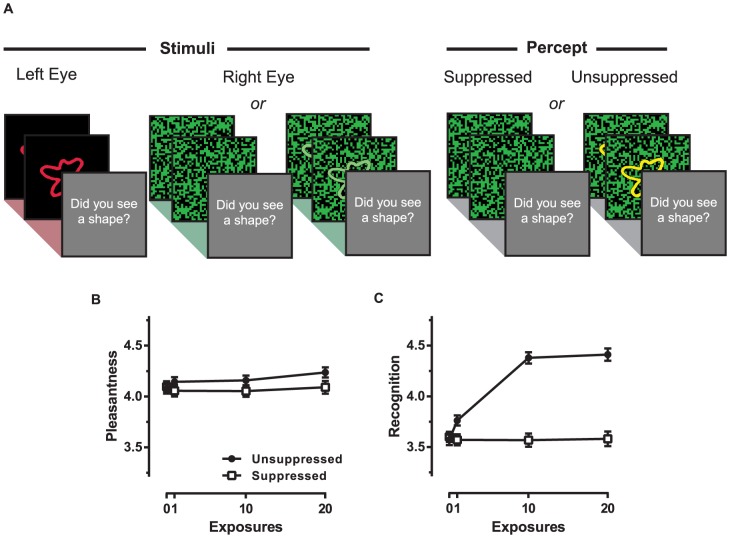
Stimuli, exposure procedure and results for suppressed and unsuppressed exposure conditions of Experiment 1. (A) Contours were presented to the left eye while either visual noise patterns or visual noise patterns with a superimposed contour were rapidly flashed to the right eye. A contour (left eye) and visual noise patterns alone (right eye) resulted in the percept of only the visual noise patterns (suppressed). A contour (left eye) and visual noise patterns with a superimposed contour (right eye) resulted in the percept of the visual noise patterns and the contour (unsuppressed). (B) Mean ratings of pleasantness for stimuli given 0, 1, 10 or 20 suppressed or unsuppressed exposures. (C) Mean ratings of recognition for suppressed and unsuppressed stimuli as a function of exposure frequency. Error bars indicate standard error of the means (SEM).

The effects of mere exposure delivered without the participant's awareness by techniques other than brief masked exposure are unknown. To thoroughly assess the role of awareness in the mere exposure effect, however, it is important to investigate different manipulations of the awareness construct. Furthermore, limitations of the brief masked exposure paradigm alone motivate the use of alternative techniques to expose stimuli without the awareness of the participant. For example, to effectively render observers unaware of the exposures, the exposures must be very brief and very closely followed by a mask [12]. When comparing these exposures to exposures with awareness using this technique, the exposure duration is confounded with awareness. It seems reasonable to suggest that exposure duration, independent of awareness, might also have consequences for the liking of stimuli; for example longer exposure durations might produce a stronger mere exposure effect by allowing more processing of the stimuli [17] or they could produce a weaker mere exposure effect by inducing boredom [8]. One technique without this duration-awareness confound, called continuous flash suppression (CFS), has proven reliable for exposing stimuli for longer durations without the subject's awareness [18]. In CFS, a target stimulus is presented to one eye while a dynamic series of visual noise or Mondrian patterns are continuously flashed to the other eye (Figure 1a). The target stimulus and the visual noise patterns compete for perceptual dominance, but because the visual noise patterns are “high-energy” the stimulus presented to the other eye can be reliably suppressed for close to one minute [18].

Another advantage of CFS is that while the stimuli can be exposed without the subject's conscious awareness, many other processing consequences remain intact. For example, functional brain imaging has demonstrated that suppressed stimuli are processed in early to high-level brain areas, from the dorsal stream of visual processing [19] to the amygdala [20], [21], [22]. Furthermore, several cognitive capabilities, such as priming [23], Pavlovian conditioning [24] and perceptual learning [25] have been shown to continue even when the stimuli critical for these capabilities are supressed from awareness. The level of processing afforded by CFS is a highly controversial matter. Other studies have shown that certain cognitive capabilities such as high-level face adaptation, but not low-level face adaptation, are removed by CFS [26], (see also [27], [28], [29]). Overall, there is evidence to demonstrate that even without visual awareness some perceptual, emotive and cognitive processes continue – demonstrating that CFS apparently allows information to reach many high-level brain structures.

A second major difficulty when interpreting the mixed results in the mere exposure literature is whether or not subjects are aware of the stimulus at the moment of exposure. Post-exposure recognition performance (ratings or forced-choice selection) is almost exclusively used as an index of awareness [9], [10], [11], [12], [15], [16]. However, a post-exposure recognition test is unlikely to be the most sensitive measure of awareness due to the passage of time between stimulus exposure and test and the interpolation of other test phases. Thus, a post-exposure recognition test is a direct measure of long-term memory, but an indirect measure of awareness at the time of the event. An insensitive and indirect awareness measure will lead to misclassification of some conscious stimulus exposures as having occurred without unawareness. Thus, to be confident that participants are not aware of exposed stimuli, awareness needs to be monitored on a trial-by-trial basis [30], [31], [32], (see also [33]).

The present study uses a technique new to the mere exposure literature to investigate the role of awareness in the mere exposure effect. The technique has two main advantages over previous methods: it uses the same exposure duration for both aware and unaware conditions; and, to maximise the sensitivity of the awareness measure, on each exposure trial subjects were asked to report their state of awareness for the exposed stimulus. In the subsequent test phases, subjects rated the suppressed, unsuppressed and similar novel stimuli on pleasantness and recognition.

To preview the results, using a highly controlled awareness manipulation and controlling the exposure duration, the mere exposure effect was found only when participants were aware of the exposed stimuli. No mere exposure effect occurred following exposures delivered outside of awareness for two different sets of stimuli.

## Experiment 1

### Method

#### Ethics statement

The study was approved by the UNSW Human Research Ethics Advisory Panel (Psychology) and conferred to the principles of the declaration of Helsinki. Participants provided written informed consent.

#### Participants

A convenience sample of all students (*N* = 1005) who were enrolled in an introductory psychology subject at the University of New South Wales participated in the experiment as part of a tutorial demonstration. Participants were tested in several groups of approximately 25 students. Participants were not screened for corrected-to-normal vision; however, only data from a subset of the sample that performed sufficiently well on a trial-by-trial awareness measure (described below) were analysed.

#### Apparatus and stimuli

The target stimuli (see Figure 1) were 16 different meaningless contours, which were coloured red (8-bit, RMS contrast 2.30 pixel units) or green (8-bit, RMS contrast 11.30 pixel units) for the exposure phase and white for the test phase. Dynamic visual noise patterns (minimum square size 7 × 7 pixels; 6-Hz) were presented to one eye the contours were presented to the other. Half of the visual noise patterns were green and black and the other half were red and black. A unique noise pattern was presented on each CFS frame.

The contours and visual noise patterns (300 × 300 pixels) were presented centrally on a 23” LCD computer monitor (1920 × 1080 resolution; 60-Hz refresh rate) and were viewed by subjects through anaglyph glasses with red (left eye) and green (right eye) filters.

#### Design and procedure

The experiment consisted of one exposure phase and two test phases (one for liking and one for recognition). At the beginning of the exposure phase, subjects were told that they would see visual noise patterns, and that sometimes a contour would be shown with the patterns. Participants were shown example contours that were not used in any other part of the experiment.

For each participant, the 16 contours were randomly divided such that eight were exposed with awareness and eight were exposed without awareness. Within each set of eight stimuli, two stimuli were randomly allocated to each of the four exposure frequencies: 0, 1, 10 and 20. The suppressed and unsuppressed contours were exposed 0, 1, 10 or 20 times in an intermixed sequence, for a total of 124 trials.

Examples of suppressed and unsuppressed exposure trials are shown in Figure 1a. For both kinds of exposure trial, a contour and a series of six visual noise patterns were exposed for one second. On suppressed exposure trials, a contour was presented to the left eye while visual noise patterns were flashed rapidly to the right eye. Perceptual dominance of the visual noise patterns typically results in the awareness of the visual noise patterns and not the contour. On unsuppressed exposure trials, a contour was presented to the left eye and the contour superimposed on visual noise patterns were presented to the right eye. Because the contour was presented to both eyes, the contour is not subject to competition for perceptual dominance. Thus, unsuppressed conditions typically result in the percept of a clearly visible contour. It is worth noting that both our suppressed and unsuppressed conditions contained the dynamic visual noise patterns. Anaglyph glasses with red and green lenses controlled the presentation of the contours and visual noise patterns to right and left eyes: contours and visual noise patterns that were intended to be presented to the right eye (green lens) were restricted to the green RGB channel and contours and visual noise patterns that were intended to be presented to the left eye (red lens) were restricted to the red RGB channel. The colour of the contours and visual noise patterns were counterbalanced for each participant.

Following each exposure, subjects completed an awareness test. Subjects were prompted to report whether they did see (left control key) or did not see (right control key) a contour using the keyboard. The subsequent trial proceeded one second after a response. We note that with this kind of awareness task alone it is impossible to identify any bias to respond that a contour was or was not seen. One concern is that subjects might adopt a conservative criterion and report that they did not see suppressed contours for which they had partial awareness. This would be particularly problematic if we were to find a mere exposure effect in the absence of awareness. While a forced-choice test between an exposed (with or without awareness) and non-exposed stimulus would allow the calculation of bias over a number of exposure trials, it is unclear how a participant's overall bias would inform the experimenter about their status of awareness on a trial-by-trial basis. Another problem with measuring awareness using a forced-choice test on a trial-by-trial basis is that such a test would constitute exposure and so would contaminate our pleasantness and recognition test phases. While still not ideal, the present study mostly relied on the accuracy of subjects' reports on the trial-by-trial awareness test to index awareness. However, to help identify subjects with a bias to report that they saw a contour, eight “catch” trials in which only a visual noise pattern (four red and four green) were presented (intermixed with the 62 suppressed trials and 62 unsuppressed trials). These “catch” trials determined the accuracy of responding when no contour was seen. If a participant repeatedly reported that a contour was present on a “catch” trial it would suggest that they had a bias towards reporting that they did see a contour.

Following the exposure phase, subjects used the keyboard to rate the pleasantness of the 16 contours, one at a time, on a seven-point scale with “highly unpleasant” and “highly pleasant” labelled at its endpoints. Similarly, subjects reported their recognition of the contours with “certain that I *did not* see it before” and “certain that I *did* see it before” at the endpoints of the seven-point scale. The experiment did not limit subjects' time to record their responses.

Data were excluded from the results based on performance on the awareness test in the exposure phase. Specifically, participants were excluded if they repeatedly reported that they saw a contour on the “catch” trials (i.e. when no contour was presented). An a priori level for exclusion according to the “catch” trials was set at less than 75 percent correct, which resulted in 73 exclusions (7% of the sample). Additionally, if a participant reported on any trial that they saw a contour that should have been suppressed, that contour was excluded from analysis. Conversely, if on any trial a participant reported that they did not see a contour that should have been visible, that contour was excluded. Thus, with the remaining data, we can be reasonably confident that suppressed contours were unseen for each of their 1, 10 or 20 exposures and unsuppressed contours were seen for each of their exposures. After the catch trial and awareness exclusions, there were 597 participants with data for each of the exposure frequencies when the contour was suppressed and 842 participants with data for each of the exposure frequencies when the contour was unsuppressed.

### Results and Discussion

To investigate the mere exposure effect with and without awareness we plotted mean liking ratings as a function of stimulus exposure frequency for both the suppressed and unsuppressed stimuli (Figure 1b). The solid black data points show pleasantness as a function of exposure frequency for stimuli that subjects had previously been aware of, while the unfilled data points display data without such awareness. Although this experiment used a within-subjects design, our method to exclude participants left an unequal number of participants who were previously unaware of the stimuli (*n* = 597) and who were previously aware of the stimuli (*n* = 842). This meant that it was not possible to perform a within-subjects analysis on these data with awareness as a factor. Thus, we first performed linear contrasts on exposure frequency separately for participants who were aware of the stimuli and for participants who were unaware of the stimuli. Linear contrasts showed a small increase in pleasantness with increased exposure frequency for unsuppressed stimuli, *F*(1,841) = 5.79, *MSE* = 1.69, *p* = .02, *d* = .17, but no increase in pleasantness with exposure frequency for suppressed stimuli, *F*(1,596) = .08, *MSE* = 1.38, *p* = .78, *d* = .02. To directly compare the effect of exposure with awareness and without awareness, we limited the analysis to participants (*n* = 544) who had data for suppressed stimuli and unsuppressed stimuli at each of the exposure frequencies. In other words, participants were excluded if they had complete unsuppressed data but incomplete suppressed data, and vice versa. This allowed us to test for an interaction between awareness and exposure frequency, or a difference in the size of linear trends for stimuli exposed with awareness compared to without awareness. With the analysis limited to these data, averaged across stimuli exposed with and without awareness there was no mere exposure effect, *F*(1,544) = .08, *MSE* = 1.73, *p* = .75, *d* = .02. Furthermore, awareness did not interact with exposure frequency and thus the sizes of the mere exposure effect with awareness and without awareness did not reliably differ, *F*(1, 544)  =  1.44, *MSE* = 1.26, *p* = .23, *d* = .10.

A recognition test was included to investigate the relationship between exposure awareness and subsequent recognition memory. Figure 1c shows increases in recognition with increased unsuppressed exposures, but not suppressed exposures. These impressions were supported by a significant linear contrast for unsuppressed stimuli, *F*(1,841) = 176.72, *MSE* = 2.42, *p*<.01, *d* = .82, but not for suppressed stimuli, *F*(1,596) = .74, *MSE* = 2.04, *p* = .39, *d* = .07. As with the pleasantness data, we conducted a further analysis on participants who had complete data for suppressed and unsuppressed stimuli thereby allowing a direct comparison between the two. With these data from suppressed and unsuppressed stimuli there was a significant increase in recognition with exposure frequency, *F*(1,544) = 49.20, *MSE* = 3.20, *p*<.01, *d* = .60, which was driven by an interaction between awareness and exposure frequency, *F*(1,544) = 82.30, *MSE* = 1.44, *p*<.01, *d* = .78. Follow-up linear contrasts showed that recognition increased with exposure for unsuppressed stimuli, *F*(1,544) = 107.88, *MSE* = 2.54, *p*<.01, *d* = .89, but not for suppressed stimuli, *F*(1,544) = .65, *MSE* = 2.09, *p* = .42, *d* = .07.

Because our online awareness measure allowed us to identify stimuli that broke suppression, we were able to investigate the effect of exposure to stimuli that were unsuccessfully suppressed and, thus, broke into awareness. These data therefore contrast with those of the above analyses, which included stimuli that were successfully suppressed for all of their exposures. We correlated the number of suppression breaks with pleasantness and recognition ratings for each stimulus (6030 in total). There was a small but significant positive correlation between number of suppression breaks and pleasantness ratings, *r* = .04, *p*<.01, and between the number of suppression breaks and recognition ratings, *r* = .10, *p*<.01.

Experiment 1 found a small mere exposure effect when participants were aware of the stimuli, but no reliable difference between stimuli exposed with awareness versus without awareness. Thus, we can make no strong conclusion about awareness from this experiment alone. The mere exposure effect is a highly robust phenomenon [34] and the effect size is normally greater than the one observed [35]. We hypothesised that one reason for our small effect size (and the failure to detect a difference between stimuli exposed with awareness versus without awareness) was that the contour stimuli used in Experiment 1 were difficult to discriminate. Thus, Experiment 2 used face stimuli, which are known to produce reliable mere exposure effects under standard procedures [14], [34], [35]. Furthermore, there is evidence to suggest that face stimuli are processed in high-level brain areas even when suppressed by CFS [20], [21], [22].

## Experiment 2

### Method

The method of Experiment 2 was the same as Experiment 1 in all aspects other than those described below. The 16 contour stimuli used in Experiment 1 were replaced with 16 faces taken from the Radboud Faces Database [36]. The faces were coloured red (8-bit, RMS contrast 11.25 pixel units) or green (8-bit, RMS contrast 5.19 pixel units) and were superimposed on the same visual noise used in Experiment 1.

There were three facial expressions for each of the 16 faces; neutral, angry, and happy. Facial expression was a between-subjects factor. Participants were randomly allocated to one of the facial expression conditions at the beginning of the experiment. Within each facial expression condition, eight faces were given suppressed exposure and eight faces were given unsuppressed exposure. Within each awareness condition, two faces (one male and one female) were allocated to each of the four exposure frequencies (0, 1, 10, and 20).

The experiment used a convenience sample; 240 second year psychology students from the University of New South Wales who participated in the experiment as part of a classroom demonstration. Participants were tested in several groups of approximately 25 students. No students who completed Experiment 1 were eligible for Experiment 2.

Experiment 2 applied the same “catch” trial and trial-by-trial awareness exclusions as did Experiment 1. Analysis of the “catch” trials resulted in 27 exclusions (11%). After the trial-by-trial awareness exclusion there were 78 participants (*n_neutral_* = 11, *n_angry_* = 38, and *n_happy_* = 29) with data for each of the exposure frequencies when the face was suppressed and 188 participants (*n_neutral_* = 44, *n_angry_* = 76, and *n_happy_* = 68) with data for each of the exposure frequencies when the face was unsuppressed.

### Results and Discussion

**Figure 2:**
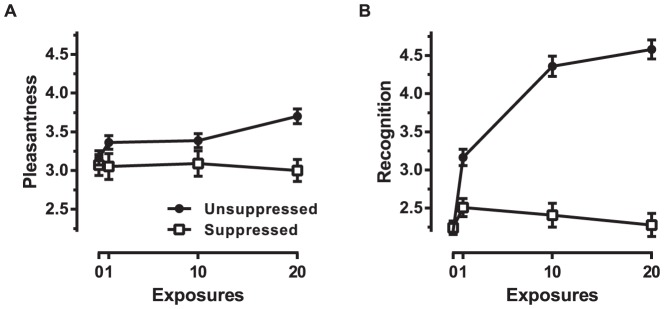
Mean pleasantness (A) and recognition (B) for faces given 0, 1, 10 or 20 suppressed or unsuppressed exposures. Error bars indicate ±SEM.

The mean pleasantness ratings as a function of stimulus exposure frequency for both suppressed (unfilled data points) and unsuppressed (solid black data points) faces are shown in Figure 2. Collapsed across all facial expressions, linear contrasts showed an increase in pleasantness as a function of exposure frequency for unsuppressed faces, *F*(1,185) = 34.83, *MSE* = .75, *p*<.01, *d* = .86, whereas the suppressed faces showed no increase in pleasantness with increased exposure frequency, *F*(1,75) = 0.08, *MSE* = .61, *p* = .93, *d* = .06. For unsuppressed faces, facial expression interacted with exposure frequency such that the mere exposure effect was larger for neutral facial expressions than both happy, *F*(1,185) = 4.02, *MSE* = .75, *p* = .046, *d* = .29, and angry, *F*(1,185) = 4.85, *MSE* = .75, *p* = .03, *d* = .32, which had similar sized mere exposure effects, *F*(1,185) = .03, *MSE* = .75, *p* = .86, *d* = .03. Follow-up linear contrasts showed that the mere exposure effect with awareness was present for neutral, *F*(1, 43) = 20.19, *MSE* = .83, *p*<.01, *d* = 1.37, happy, *F*(1,67) = 6.28, *MSE* = .84, *p* = .02, *d* = .61, and angry, *F*(1,75) = 7.94, *MSE* = .62, *p*<.01, *d* = .65, facial expressions. Facial expression for suppressed faces did not interact with exposure frequency, highest *F*(1,75) = 2.09, *MSE* = .61, *p* = .15, *d* = .33. As in Experiment 1, we directly compared stimuli that were exposed with the awareness of the subject to stimuli exposed without awareness. An additional motivation for this analysis was the greater number of subjects in the unsuppressed condition compared to the suppressed condition (which increased our likelihood of finding a mere exposure effect with awareness). Thus, we tested for an interaction between awareness and exposure frequency for participants who had complete data for both the suppressed and unsuppressed stimulus conditions, thereby equating the number of subjects (*N* = 65). In these conditions, there was no linear trend for exposure frequency averaged across faces exposed with awareness and without awareness, *F*(1,64) = 2.17, *MSE* = .82, *p* = .15, *d* = .37. However there was a reliable interaction between awareness and exposure frequency, *F*(1,64) = 6.21, *MSE* = .49, *p* = .01, *d* = .63. Follow-up contrasts confirmed that there was a significant linear trend for faces exposed with awareness, *F*(1, 64) = 7.08, *MSE* = .67, *p* = .01, *d* = .67, and not for faces exposed without awareness, *F*(1, 64) = 0.14, *MSE* = .64, *p* = .72, *d* = .09.

Mean recognition as a function of stimulus exposure frequency for suppressed and unsuppressed faces are shown in Figure 2b. Recognition increased with exposure frequency for unsuppressed faces, *F*(1, 185) = 284.78, *MSE* = 2.24, *p*<.01, *d* = 2.47, but not suppressed faces, *F*(1, 75) = .03, *MSE* = 1.59, *p* = .86, *d* = .04. Facial expression did not interact with exposure frequency for suppressed faces, highest *F*(1, 75) = 1.97, *MSE* = 1.59, *p* = .17, *d* = .29, or unsuppressed faces, highest *F*(1, 185) = 3.86, *MSE* = 2.24, *p* = .051, *d* = .29. The pattern was still reliable when there were equal numbers of subjects (*N* = 65) in unsuppressed and suppressed conditions. Across faces exposed with and without awareness, exposure increased recognition, *F* = 70.77, *MSE* = 1.95, *p*<.01, *d* = 2.10. The effect seems to be driven by an interaction between awareness and exposure frequency, *F*(1, 64) = 60.68, *MSE* = 1.63, *p*<.01, *d* = 1.95. Follow-up contrasts showed that exposure increased recognition when participants were aware of the faces, *F*(1, 64) = 137.17, *MSE* = 1.72, *p*<.01, *d* = 2.93, but not when they were unaware of the faces, *F*(1, 64) = .87, *MSE* = 1.87, *p* = .35, *d* = .23.

As another measure of the mere exposure effect in the presence of awareness, we correlated the number of suppression breaks for each stimulus (1440 in total) with pleasantness and recognition ratings. There was a small but significant positive correlation between the number of suppression breaks and pleasantness ratings, *r* = .14, *p*<.01, and a stronger positive correlation between the number of suppression breaks and recognition ratings, *r* = .57, *p*<.01.

The experiment replicates our original finding with face stimuli and shows a notably larger effect of exposure frequency on pleasantness and on recognition. Thus, the data are consistent with our hypothesis that the small mere exposure effect with awareness in Experiment 1 was due to the use of stimuli that were difficult to discriminate. More discriminable (face) stimuli were sufficient to increase the size of the mere exposure effect with awareness, yet no mere exposure effect was observed without awareness.

## General Discussion

The results of the present study are consistent with the idea that awareness is necessary for the mere exposure effect. Using a highly controlled manipulation of awareness and holding stimulus duration constant, our data suggest that stimuli exposed without awareness are not liked any more than novel stimuli. In contrast, familiarity with stimuli via conscious exposure leads to increased liking of those stimuli.

Our conclusion rests on the assumption that suppressed stimuli are processed in some manner, but that this processing is insufficient to “break into” awareness or lead to a mere exposure effect. The evidence for this crucial assumption comes from neuroimaging studies that show suppressed stimuli are processed in high-level brain areas (e.g. [19], [20], [21], [22]) and from behavioural data that show exposure to suppressed stimuli has many cognitive effects (e.g. [23], [24], [25]). However, at what level of neural processing our stimuli are being processed without awareness remains a controversial question. We know with confidence that our stimuli are reaching neural populations in the early visual cortex [37], beyond this it becomes hard predict. A minority of published behavioural studies might lead us to make a different assumption – that only low-level visual properties are processed during suppression (e.g. [26], [27]), or at least that high-level effects of suppressed exposure are relatively small. Thus, if constrained by this alternative view our conclusion would be simply that the mere exposure effect does not exist when limited to low-level visual processing that is outside of conscious awareness. Furthermore, this alternative conclusion invites the possibility that the mere exposure effect exists following suppression from awareness that is weaker or later than suppression produced by CFS, for example via brief masked exposure [23]. Experiments targeted at determining which of these two accounts provides the more accurate interpretation of our data are a clear goal for future research.

The present results are consistent with past studies that show a relationship between familiarity and liking. Newell and Shanks [14] found no mere exposure effect when participants were made unaware of the stimuli via brief masked exposure. However, they did find a mere exposure effect when participants were both aware of the stimuli at exposure and remembered that the stimuli had been exposed at test (for a similar finding, see [38]). Together with our finding that exposure with awareness increases liking and recognition, the data suggest that the mere exposure effect relies on explicit memory. Our results, especially those with emotional faces in Experiment 2, also supports evidence outside the mere exposure literature that suggests preferences are unaltered by exposure via CFS. Specifically, previous evidence suggests that CFS (and brief masked exposure), although they might allow low-level processing such as perceptual priming, disrupt the processing of valence from which preferences might arise [39].

The present data contrast with past studies that propose the mere exposure effect occurs without awareness (e.g. [2], [12]), and furthermore, with past results interpreted to suggest that the absence of awareness increases the exposure effect [9], [35]. The different conclusions might be attributable to differences in the level of suppression afforded by CFS and by brief masked exposure. Nevertheless, two limitations of previous studies may also have an influential role in producing the mere exposure effect in the absence of awareness. Firstly, whereas past studies have used a brief exposure paradigm with no test of awareness (e.g. [2]) or with a post-exposure recognition test (e.g. [9], [12]), the present experiments used an “online” trial-by-trial test of awareness. The results of the “online” trial-by-trial awareness test demonstrated that 36 percent of our sample in Experiment 1 and 64 percent of our sample in Experiment 2 was aware of a supposed suppressed stimulus. None of these participants would have been excluded on the basis of a traditional post-exposure recognition test alone. Thus, not removing these participants or stimuli would lead to an error in which exposure of which the subject is aware at the time of exposure is misclassified as unaware post-exposure. The consequence is a bias to classify stimuli as exposed outside of awareness. Although differences in procedure make it difficult to estimate the extent of any misclassification in previous studies, it is possible (perhaps likely) that aware participants who were misclassified as unaware contributed to exposure effects thought to have occurred outside of awareness. Furthermore, our data supports this possibility by showing that stimuli that break into awareness during the CFS procedure show mere exposure effects.

The second relevant limitation of past studies is the covariation of awareness and exposure duration. The exposure technique used in previous studies resulted in exposures with awareness and exposures without awareness receiving unequal exposure durations. Importantly, exposure duration seems to independently influence the size of the mere exposure effect. Bornstein's [35] meta-analysis shows stronger exposure effects with shorter exposure durations; specifically, exposure durations of less than one second produced stronger effects than exposures of one or more seconds. Assuming that this trend is monotonically decreasing, any finding of a stronger mere exposure effect following very brief exposure (e.g. 5 ms) compared to longer exposure (e.g. 500 ms), such as Bornstein & D′Agostino [9], can be interpreted as a consequence of unequal exposure duration rather than an interpretation specifically regarding awareness. Our results favour the former interpretation because they question whether the absence of awareness *per se* can lead to a strong mere exposure effect; on the contrary, when the exposure duration was equal, we found a mere exposure effect with awareness and no mere exposure effect without it.

Supposed exposure effects without awareness have previously been taken to support a class of theories that suggest the exposure effect is inhibited when the increase in pleasantness as a consequence of exposure can be easily attributed to exposure [9], [40]. For example, Bornstein & D′Agostino [41] found that the exposure effect is weaker when participants are informed that all the test stimuli were previously exposed (therefore encouraging such an attribution), and stronger when participants are informed that all of the test stimuli were novel (therefore discouraging such an attribution). Similarly, longer exposure durations might lead to weaker effects than brief exposure durations because it is easier to attribute an increase in pleasantness to exposure when the exposure is more obvious or salient (see [42]). On the one hand, the claims of these theories regarding the attribution of pleasantness to exposure and subsequent discounting of pleasantness remain largely unchallenged by the present work; in certain situations weaker attributions lead to stronger mere exposure effects [41], [42]. However, the caveat that the present research provides is that when pleasantness cannot be attributed to exposure because of (possibly strong) suppression of the stimuli from the participant's awareness, there is no mere exposure effect.

Using continuous flash suppression and controlling stimulus duration, we were twice able to replicate the mere exposure effect with awareness (thus demonstrating that this novel technique is suitable for obtaining the standard effect) but found no evidence of a mere exposure effect without awareness. The result highlights limitations of previous research that has relied on rendering stimuli invisible using a brief exposure paradigm, yet we note that inconsistent results within the brief exposure paradigm and between the brief exposure paradigm and the present CFS paradigm are far from resolved. Based on this empirical result with the CFS paradigm and previous inconsistent results with the brief exposure paradigm, we question previous claims that increased liking following brief exposure demonstrates the effects of an implicit memory system, and that the brief exposure paradigm shows evidence of separate affective and cognitive systems. Rather, we show that liking and recognition are similarly affected by aware exposure and unaffected by unaware exposure. Thus, our data are consistent with the idea that increases in liking and recognition following exposure result from a common explicit memory source.
